# Synchronous LC-MS/MS determination of pantoprazole and amitriptyline in rabbit plasma: application to comparative in vivo pharmacokinetic study of novel formulated effervescent granules with its marketed tablet dosage form

**DOI:** 10.1016/j.heliyon.2021.e07752

**Published:** 2021-08-10

**Authors:** Asmaa A. El Zaher, Ehab F. El Kady, Hussein M. EL Messiry, Hind E. El Ghwas, Ola M. El Houssini

**Affiliations:** a Pharmaceutical Chemistry Department, Faculty of Pharmacy, Cairo University, Kasr El-Aini St., Cairo 11562, Egypt; b Pharmaceutics Department, National Organizations for Drug Control and Research (NODCAR), 51 Wezerat El- Zeraa Street, Agouza, P.O. Box 12553 Giza, 35521, Egypt; c Pharmaceutical Chemistry Department, National Organizations for Drug Control and Research (NODCAR), 51 Wezerat El- Zeraa Street, Agouza, P.O. Box 12553 Giza, 35521, Egypt

**Keywords:** LC-MS/MS, Pantoprazole, Amitriptyline, Effervescent granules, Bioanalysis, Pharmacokinetics

## Abstract

In the present study the bioavailability and pharmacokinetics properties of pantoprazole (proton pump inhibitor)/amitriptyline (tricyclic antidepressant) in novel formulated effervescent granules was estimated in rabbit plasma using a validated, selective and rapid LC-MS/MS method. Separation and detection of pantoprazole, amitriptyline and internal standards namely omeprazole and dothiepin, respectively, were achieved at ambient column temperature on C_18_. Acetonitrile: 4mM ammonium acetate solution (comprising 0.05 % formic acid) (40:60, v/v) was used as mobile phase and the flow rate of 0.6 mLmin^-1^ was applied. Liquid-liquid extraction technique with diethyl ether: dichloromethane (70:30, v/v) was used to extract the cited drugs from rabbit plasma. Multiple reactions monitoring (MRM) in the positive ionization mode was carried out for quantification. The method was validated over linear concentration range of 0.01-4μgmL^−1^ and 0.001–0.1 μgmL^−1^ for Pan and Ami respectively, with regression coefficient (r^2^) ≥ 0.9961. The intra- and inter-run precisions (%CV) were ≤4.03. The extraction recoveries were in the range of 95.92%–100.24 %. Pan and Ami were stable during three freeze-thaw cycle and post-preparative stability. The work also aimed to formulate immediate release novel effervescent granules by melt granulation technique. Nine formulae were assessed by validated dissolution test for their micrometric properties and dissolution profile. Experimental design was applied to select formula that fulfilled the desired criteria of optimum release of pantoprazole and amitriptyline with optimum micrometric properties for the study. A single period randomized open-label parallel design was applied on Chancellor's rabbit. The selected formula showed superior pharmacokinetic parameters for pantoprazole and amitriptyline than that of marketed products.

## 1 Introduction

**Figure 1:**
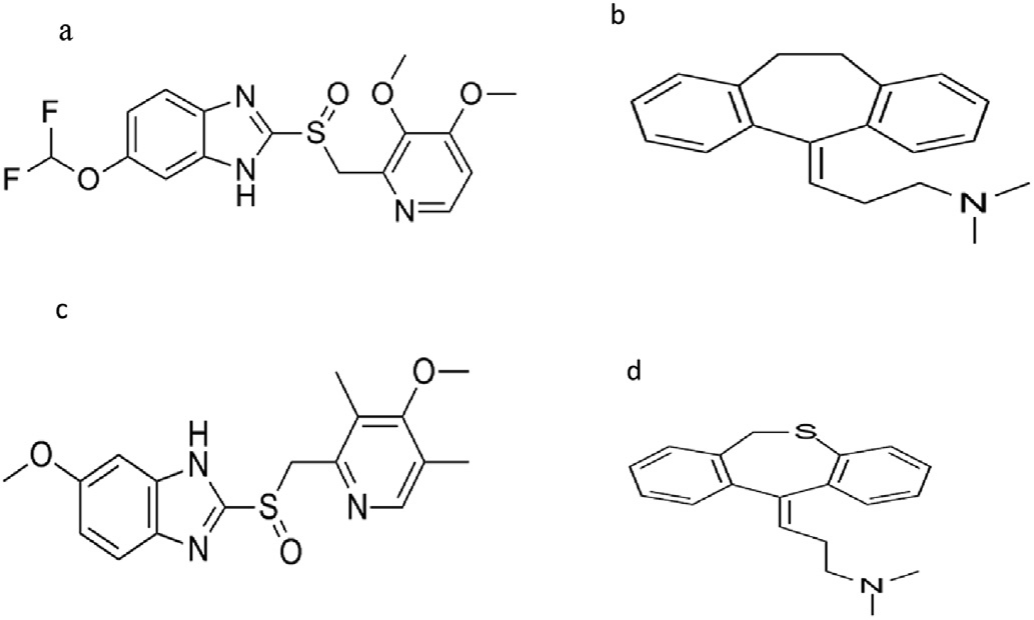
Chemical structure of (a) Pan, (b) Ami, (c) Omp and (d) Dot.

Pantoprazole, 5-(difluoromethoxy)-2-[(3, 4-dimethoxypyridin-2-yl) methylsulfinyl]-1H-benzimidazole (Pan, Figure 1a) (O'Neil, 2006), a proton pump inhibitor (PPI), is unstable in acidic solutions and undergoes rapid acid-catalyzed degradation, while it shows stability at neutral or alkaline pH. Due to its pH sensitivity, effective drug delivery is problematic. The inhibition of H+/K^+^-ATPase in the gastric parietal cell by PPIs, results in suppressing gastric acid secretion (Roche, 2006). Most PPIs are formulated as an enteric-coated solid dosage forms. The dissolution of enteric-coated dosage form varies from one individual to another as it is affected by gastric emptying time, variability of pH of gastrointestinal tract and other physiological factors such as the fed or fasted state. Accordingly, the variability of dissolution times of this dosage form leads to variability of their pharmacokinetic profiles between individuals (Taneja et al., 2009; Rajneesh et al., 2009; Guo et al., 2011).

Amitriptyline, 3-(10,11-dihydro-5H-dibenzo[a,d] cycloheptene-5-ylidene)-N,N-dimethylpropan-1-amine (Ami, Figure 1b) (O'Neil, 2006), a tricyclic antidepressant (TCA) utilized in management of fibromyalgia which may be complex chronic disorder distinguished by physical fatigue, diffused pain, non-restorative sleep and cognitive impairment (Borchers and Gershwin, 2016; Lawson, 2016).

It was found that depression is a stress-related mood disorder characterized by depressive cognition and emotional dysregulation of individuals (Chen et al., 2019). It is a potential contributor to development of peptic ulcer (PU) and gastro esophageal reflux disease (GERD) with recurrence rate in depressed individual higher than non-depressed ones (Chen et al., 2019; Oh et al. ., 2009). For that Anxipan® capsule, a combination of Pan as a main active ingredient and Ami which reduces the depression symptoms that may contribute to the development of PU was recently introduced in Indian market. Several studies had been reported in literature determining the cited drugs separately in human plasma or in combination with other drugs by HPLC method (Linden et al., 2008; Farag et al., 2013; Nayanda et al., 2000; Ali et al., 2016) or by LC-MS/MS in human plasma Table 1S (Peres et al., 2004; Li et al., 2011; Challa et al., 2010; Kudo et al., 1997; Elkady et al., 2018; Breaud et al., 2010; Sauvage et al., 2006) and in human's urine (Bhaskara et al., 2011). But none of them focused on the bioequivalence between two formulations or studying the pharmacokinetics parameters of Pan and Ami together in one formula.

Referring to the above mentioned details, this study represents the first synchronous LC-MS/MS determination of Pan and Ami in rabbit' plasma. The developed method was used for the determination of pharmacokinetic parameters of Pan and Ami in combined innovative formula (Treatment A) and commercial tablets (Treatment B) of the same strength available in Egyptian pharmaceutical market (Tryptizol® oral tablets; Amitriptyline HCl 10mg) (Kahira for pharmaceutical industry, Egypt) and (Zurcal® gastro-resistant tablets; Pantoprazole 40mg) (AUG pharma, Egypt).

The innovative formula (Treatment A) was selected depending on its micrometric properties and dissolution profile. As dissolution test provides information about the product quality as well as in vitro/in vivo correlation (Barakat et al., 2015), dissolution method was developed and validated to choose the optimum formula according to the optimum release of Pan and Ami.

## 2 Experimental

### 2.1 Reagents and materials

Pantoprazole sodium sesquihydrate (Pan. SS) certified to contain 99.71 % ± 0.50, amitriptyline HCl (Ami. HCl) certified to contain 100.4 % ± 0.35; Omeprazole sodium (Omp. S, Figure 1c) certified to contain 99.50 % ± 0.25, and dothiepin HCl (Dot. HCl, Figure 1d) certified to contain 99.65 % ± 0.21 were obtained from National Organization for Drug Control and Research (NODCAR). Zurcal® tablet (40mg Pan) (Lot. No.191090), Tryptizole® tablet (10mg Ami) (Lot. No.2010811) were supplied from their companies Astera zenca and Kahira Pharmaceuticals (Cairo, Egypt) respectively. Citric acid, sodium carbonate and sodium bicarbonate (El Nasser, Egypt), polyethylene glycol 4000 (PEG 4000) and vanillin (Sisco research lab-India) were bought from local market. LC-grade solvents and all analytical grade reagents were used: Methanol and acetonitrile (Sigma-Aldrich, Germany), Formic acid (Scharlau, Spain), ammonium Acetate (Merck KaGA, Germany), sodium phosphate (Acros, USA), dichloromethane (Fisher Scientific, UK), diethyl ether (Merck KaGA, Germany). Deionized water was produced by water purifier (Pure lab option- R7ELGA, UK). Vacutainer tubes containing potassium ethylenediaminetetraacetic acid (K2EDTA) and Nylon membrane filters (0.2 μm) from Teknokroma (Barcelona, Spain) for filtration of the mobile phase were bought from local supplier.

### 2.2 Instrumentation

The Agilent 1200 series LC system (USA) fortified with Agilent 6410 triple quadruple LC-MS/MS detector (USA), quaternary gradient pump, auto-sampler, vacuum degasser and mixer was used. Agilent Mass Hunter software (B.03.01) and mass Hunter quantitative analysis software (B.04.00) were adopted for data acquisition and quantitation, respectively. Other instruments used were: pH-meter Jenway 3510 (Bibby Scientific, Felsted, Essex, UK), Vortex mixer (Boeco, Germany), analytical balance (Sartorius, USA), ultrasonic processor (Crest, USA), concentrator plus/Vacufuge® plus (Eppendorf, Germany), Hermle Labortechnik GmbH centrifuge Z326K (Wehingen, Germany), hot plate and stirrer (Jenway, Dunmow, Essex, UK) and dissolution test station (Hanson Research, SR8 Plus, Chatsworth, USA). WinNonlin® (v3) and Design –Expert® 11 software were used for Pharmacokinetics parameters calculation and to perform DOE (full factorial design for prepared formulae optimization), respectively.

### 2.3 Animals

Two equal size dosing group each consisted of six healthy male Chancellor's rabbit weighted 3.65 ± 0.07 kg were randomly numbered. The rabbits were offered by NODCAR's farm. The protocol was approved by Ethics Committee of Animal Care and use of National Organization of Drug Control and Research, Giza (Approval No І/20/19).

### 2.4 LC-MS/MS conditions

**Figure 2:**
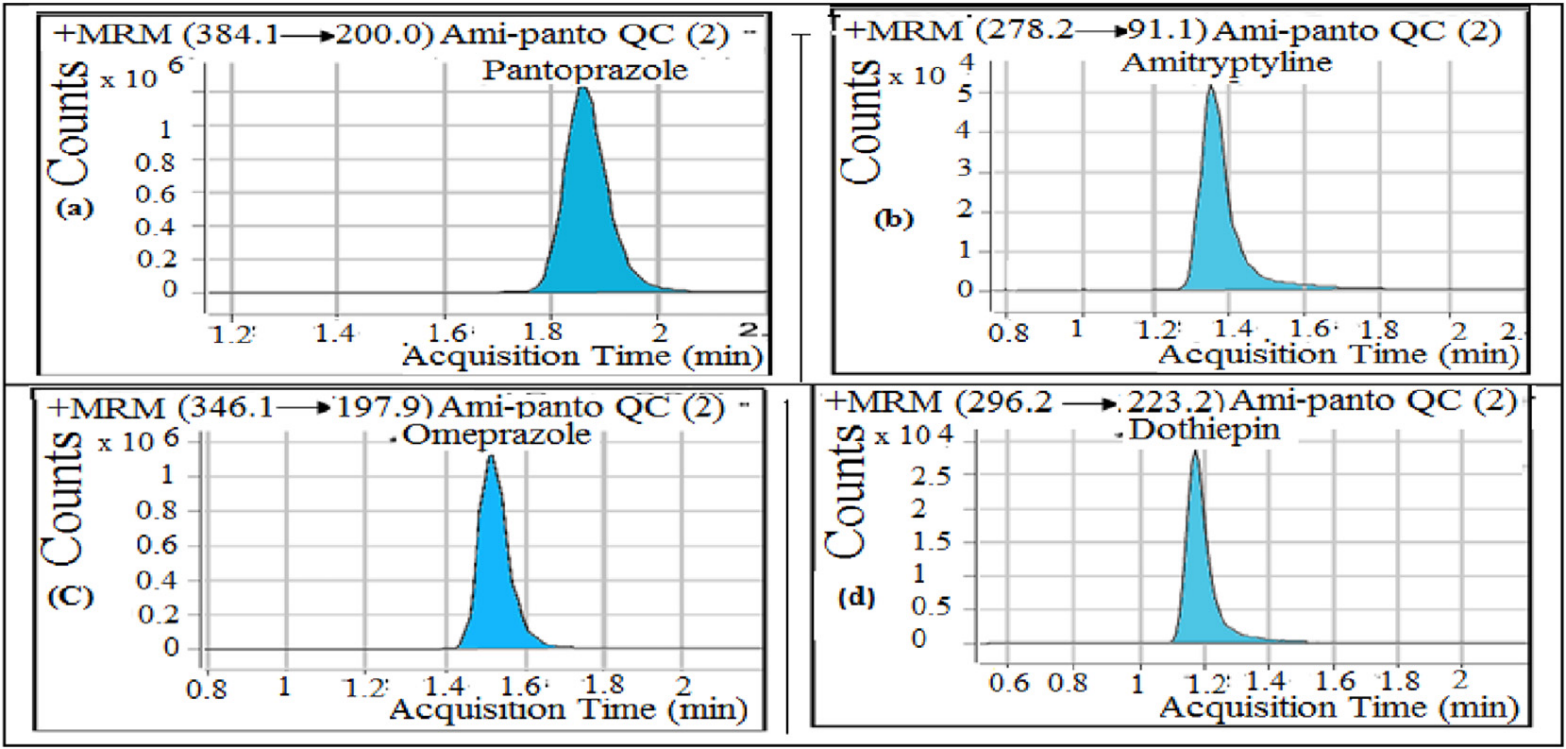
MRM chromatographic signal corresponding to medium quality control samples of: (a) Pan (1.6μgmL^−1^) at 1.85min, (b) Ami (0.03μgmL^−1^) at 1.41min, (c) Omp (3μgmL^−1^) at 1.51min and (d) Dot (2μgmL^−1^) at 1.20min.

At ambient column temperature, the chromatographic separation was performed on Gemini Phenomenex column C_18_ (4.6 × 50 mm, 5um) using acetonitrile: 4mM ammonium acetate (comprising 0.05 % formic acid) (40:60, v/v) as mobile phase pumped at a flow rate 0.6 mLmin^-1^ and 7 μL injection volume. The retention times of Pan, Ami, Omp and Dot were 1.85, 1.41, 1.51 and 1.17 min, respectively, with total run time of 2.2 min (Figure 2). The pressure of spray gas was 48 psi with nitrogen flow (10 Lmin^-1^), dwell times (150 ms) and voltage of capillary was (5000 V). Energies of collision were set at 29, 30, 28, 27 V for Pan, Ami, Omp and Dot, respectively. The fragmentor voltage for Pan and Omp was set at 90.0 V and for Ami and Dot at 130.0V. Multiple Reaction Monitoring (MRM) transitions were measured at positive mode at: m/z 384.1→200 for Pan, m/z 278.2→91 for Ami, m/z 346.1→197.9 for Omp and 296.2→223.2 for Dot.

### 2.5 Standard solutions

#### 2.5.1 For Pan and Ami

Pan and Ami stock solutions (100 μgmL^−1^) were prepared by dissolving 10 mg of each drug separately in 100 mL methanol. Then, 12.5 mL and 0.5 mL of Pan and Ami stock solutions were separately diluted to 25 mL with methanol: water (50:50, v/v), (working solutions, 50 and 2 μgmL^−1^ for Pan and Ami, respectively).

#### 2.5.2 For IS (Omp and Dot)

IS stock solutions (100 μgmL^−1^) were prepared by dissolving 10 mg of each drug separately in 100 mL methanol. Then, 7.5 mL Omp and 5 mL Dot IS stock solutions were diluted to 25 mL with methanol, (IS working solutions, 30 and 20 μgmL^−1^ for Omp and Dot, respectively).

#### 2.5.3 Calibration standard solutions and quality control solutions

Into two series of 10-mL volumetric flasks different aliquots from working solutions of Pan (50 μgmL^−1^) and Ami (2 μgmL^−1^) were accurately transferred to prepare calibration standard solutions (0.1–40 μgmL^−1^) and (0.01–1 μgmL^−1^) for Pan and Ami, respectively and quality control (QC) solutions (0.3, 16 and 30 μgmL^−1^) and (0.03, 0.3 and 0.750 μgmL^−1^) for Pan and Ami, respectively. To prepare nine non-zero samples covering the expected range of concentration (0.01–4 μgmL^−1^) and (0.001–0.1 μgmL^−1^) for Pan and Ami, respectively, 20 μL of each drug calibration standard solution were added to 160 μL blank rabbit's plasma samples and subjected to sample extraction procedure. Following the same procedure, QC samples were prepared to attain three different QC samples at different concentration levels. The final plasma concentrations of the low (LQC), medium (MQC) and high (HQC) samples were (0.03, 1.6 and 3 μgmL^−1^) and (0.003, 0.03 and 0.075 μgmL^−1^) for Pan and Ami, respectively.

### 2.6 Bio-analytical method validation

According to FDA and EMA guidelines (Food and Drug Administration, 2011; Guideline EMEA 192217, 2015) linearity, selectivity, extraction recovery, matrix effect, accuracy, dilution integrity and stability, bio-analytical method validation was conducted.

### 2.7 Sample extraction procedure

Into a series of centrifuge tubes, 20 μL from IS working solutions, then 100 uL phosphate buffer pH 11 were added to 200 μL spiked sample containing 20 μL of each Pan and Ami calibration standard or QC solutions. Phosphate buffer pH 11 is similar to the pKa of both drug (pka of Pan = 9.15 and pka of Ami = 9.7), that forced the studied drugs and IS to exist in unionized form, that enhanced good extraction recovery from the plasma and prevented Pan from acid degradation and so, increased its stability. The samples were mixed by vortex for approximately 1.5 min, then 3 mL diethyl ether: dichloromethane (70:30, v/v) were added and the samples were remixed for approximately 1–2 min. The sample was centrifuged for 5 min at 3800 rpm at 4 °C. The clear organic layer was then transferred to a clean test tube. The organic layer was evaporated at 45 °C till dryness. The residue was reconstituted with 200 μL acetonitrile: 4mM ammonium acetate solution (40:60, v/v), then clear sample was transferred to a vial insert.

### 2.8 Preparation of Pan and Ami effervescent granules

The effervescent granules were prepared by melt granulation technique (Pradeep, 2013; Agrawal and Naveen, 2011), that was achieved by adding a meltable binder of PEG4000 at concentration 5 % of the total weight granulation (Jassim et al., 2018) in solid state at room temperature. Whereas no further addition of liquid binder or water was required in the process as the binder in the molten state act as granulating liquid. Sodium carbonate (Na_2_CO_3_) and sodium bicarbonate (NaHCO_3_) were used as buffering agents that protected Pan by increasing the pH of the stomach. So, increasing its stability and lowering the rate of its degradation in stomach. The detail process was as follow:

Different amounts of (Na_2_CO_3_) and (NaHCO_3_) were added to 40 mg of Pan, 10 mg Ami, known amount of citric acid and vanillin. PEG 4000 was heated in a mortar on hot plate at temperature 40 °C till melting. The above ingredients were added into the mortar with continuous stirring till the granules were formed. The resulted granules were dried at room temperature, and placed in tightly sealed containers. General factorial experimental design (3^2^) was adopted to determine composition of the prepared formulae (Refer to supplementary material Table 2S).

### 2.9 Evaluation of the prepared effervescent granules (Aulton and Taylor, 2002; Bastos et al., 2008)

The prepared formulae were inspected for their physical character such as color, odor and homogeneity and evaluated to their micrometric properties, effervescence cessation time, pH determination and dissolution test.

### 2.10 Dissolution method validation

In vitro dissolution studies were conducted using USP (Rockville, 2019) dissolution apparatus type II paddle at 75rpm in Hausner dissolution tester. The dissolution was carried out for a total period of 60 min at 37.0 ± 0.5 °C. 1000mL of 0.1N HCl was chosen as it was adopted by USP (Rockville, 2019) in dissolution for Ami. It was also used for Pan to determine its release in stomach without degradation. From each vessel, samples were withdrawn at 5, 10, 15, 20,25,30,45 and 60min. The percentage of released drug was estimated by HPLC using mobile phase containing 0.05 M ammonium acetate: acetonitrile (55:45, v/v) with isocratic elution mode using Hypersil BDS C8 (4.6 × 250 mm, 5μm) column at room temperature, with flow rate of 1mLmin^-1^. The detection was conducted at 230 nm. According to USP guidelines for dissolution development and validation (Rockville, 2019), the method was validated with respect to accuracy precision, specificity, linearity and robustness.

### 2.11 Statistical design for the study

General factorial experimental design (3^2^) was used to study the influence of two factors (independent variables): Basic excipients (Na_2_CO_3_ and NaHCO_3_) concentrations at three levels 200, 600 and 800 mg/2000, 5800 and 8500 mg, respectively on the percentage release of Pan and Ami prepared formulae after 60 min, which were selected as (depended variables) using Design –Expert® software (Refer to supplementary material Table 3S). Desirability was then calculated to select the formula of the optimum conditions (Refer to supplementary material Table 2S).

### 2.12 Stability study

The formula with highest desirability exposed to long term stability study (The International Council for Harmonisation Q1A R2, 2003) according to ICH guidelines. The selected formula was packed in plastic container and stored at 30 ± 2 °C and 65 ± 5 % R.H (Lund, 1994). Physical inspection, micrometric properties, effervescence cessation time, pH in water and 0.1N HCl and in vitro dissolution a studies were performed every three months (i.e. at zero time and after 3, 6, 12 months).

### 2.13 Pharmacokinetic studies

#### 2.13.1 Study design and drug administration

Owing to its high sensitivity, the proposed method was applied for the pharmacokinetic study of Pan and Ami in rabbit's plasma. The selected formula (Treatment A) was chosen for bioequivalence study in comparison with commercial tablets (Treatment B) of same strength available in Egyptian pharmaceutical market.

In this protocol, a single period randomized open-label parallel design was applied and approved by the animal care committee in NODCAR in Egypt at 16/10/2018. The rabbits were divided, randomly numbered and fasted for 24 h to minimize the effects of food on pharmacokinetic profile and to allow access of water and the administered dose to rabbit. Treatments A (solution form) and B (suspension form) were administrated at 9:00am by using 20-mL polypropylene syringe. The rabbit's dose of Pan and Ami was determined by either dividing or multiplying the human dose (mg/Kg) by the (k_m_) ratio on the basis of body surface area (Guideline EMEA 133, 2017; Nair and Jacob, 2016). For analysis, 2 mL venous blood samples were withdrawn into vacutainer tubes containing potassium ethylenediaminetetraacetic acid (K2EDTA) at specified pre-determined time intervals (0, 0.167, 0.333, 0.5, 1, 1.5, 2, 2.5, 3, 3.5, 4, 6, 10, 12, 18 and 24 h) post dose. After collection, all the samples were centrifuged at 3500 rpm for 10 min at 4 °C. Then separated and stored at -70 °C till LC determination.

#### 2.13.2 Pharmacokinetic and statistical analysis of data

The following parameters were assessed for a period of 0–24 h. Maximum plasma concentration (C _max_), time of maximum plasma concentration time (T_max_), were taken directly from individual concentration versus time profiles plot. By using the linear trapezoidal method, the area under the concentration–time curve from time zero to time of last quantifiable concentration AUC _0-24_ for 0–24 h was computed. Extrapolation of AUC from baseline to infinity (AUC_0–∞_) was computed as follows: AUC_0–∞_ = AUC_0–t_ + (Ct/k_el_), Ct was the last measurable plasma concentration; Equation (1). AUMC_0-∞_, Area under the first moment curve from time zero to infinity, it is the area under the curve of concentration x time versus time from time zero to infinity (AUMC_0-∞_) _=_ ∫ ^0^_∞_ = C.tδt Equation (2). Elimination half life (t_½ el_) = 0.693/K _el_, Equation (3). Elimination rate constant (K _el_) = slope of the end of the straight part of logarithmic concentration–time curve x -2.303, Equation (4). Absorption half-life (t _½ abs_) = 0.693/K _abs_ Equation (5). Absorption rate constant (K _abs_) = slope of residual line of logarithmic concentration–time curve x -2.303, Equation (6). Apparent clearance (CL/F) = dose drug administrated (X)/AUC_0–∞_, Equation (7). Mean residual time up to infinity (MRT _**0-∞**_**) =** AUMC_0-∞_/AUC_0–∞_, Equation (8) (Persky, 2013). The pharmacokinetic parameters was calculated by WinNonlin® (v3) software and statistically evaluated by one way analysis of variance (ANOVA) using SPSS® 2000 version 7.5 software in order to investigate the statistical significance among the pharmacokinetic parameters. Statistics significance was attained at *p* value ≤0.05.

## 3 Results and discussion

### 3.1 Optimization of bioanalytical method

#### 3.1.1 Chromatographic conditions

Several columns of reversed phase were tried such as Waters Symmetry C_18_ (4.6 × 50 mm, 3.5μm) and Gemini Phenomenex column C_18_ (4.6 × 50mm, 5um). Also, various trials were carried out for mobile phase compositions with different acidified water ratios (acetic acid/formic acid), buffers (ammonium acetate/ammonium formate) with methanol/acetonitrile as organic modifiers in an isocratic elution mode. A mobile phase consisting of acetonitrile: 4.0 mM ammonium acetate solution (comprising 0.05 % formic acid) (40:60, v/v) pumped at flow rate 0.6 mLmin^-1^ on a Gemini Phenomenex C_18_ (4.6 × 50 mm, 5um) column in isocratic elution mode was applied. Ammonium acetate buffer (4mM) prohibited the endogenous component in matrix and plasma protein to interfere at the retention times of studied drugs and IS. It enhanced the extraction recovery and selectivity with acceptable IS- normalized matrix effect. So, better peak shape and highest detection response were obtained. Increasing or decreasing buffer concentration above and below this value (4mM) did not give reproducible extraction recovery and insufficient selectivity. The presence of 0.05 % formic acid in mobile increased the sensitivity of MS detection due to better positive ionization of the ions that improved peak shape. The synchronous determination of the cited drugs with diverse pK_a_ values was accomplished with required optimized response (peak area/shape). The presence of formic acid (0.05 %) in mobile phase led to increase the sensitivity of MS detection due to better positive ionization of the ions. Appling these conditions, the run time of 2.2 min was accomplished.

#### 3.1.2 Mass spectrometric conditions

**Figure 3:**
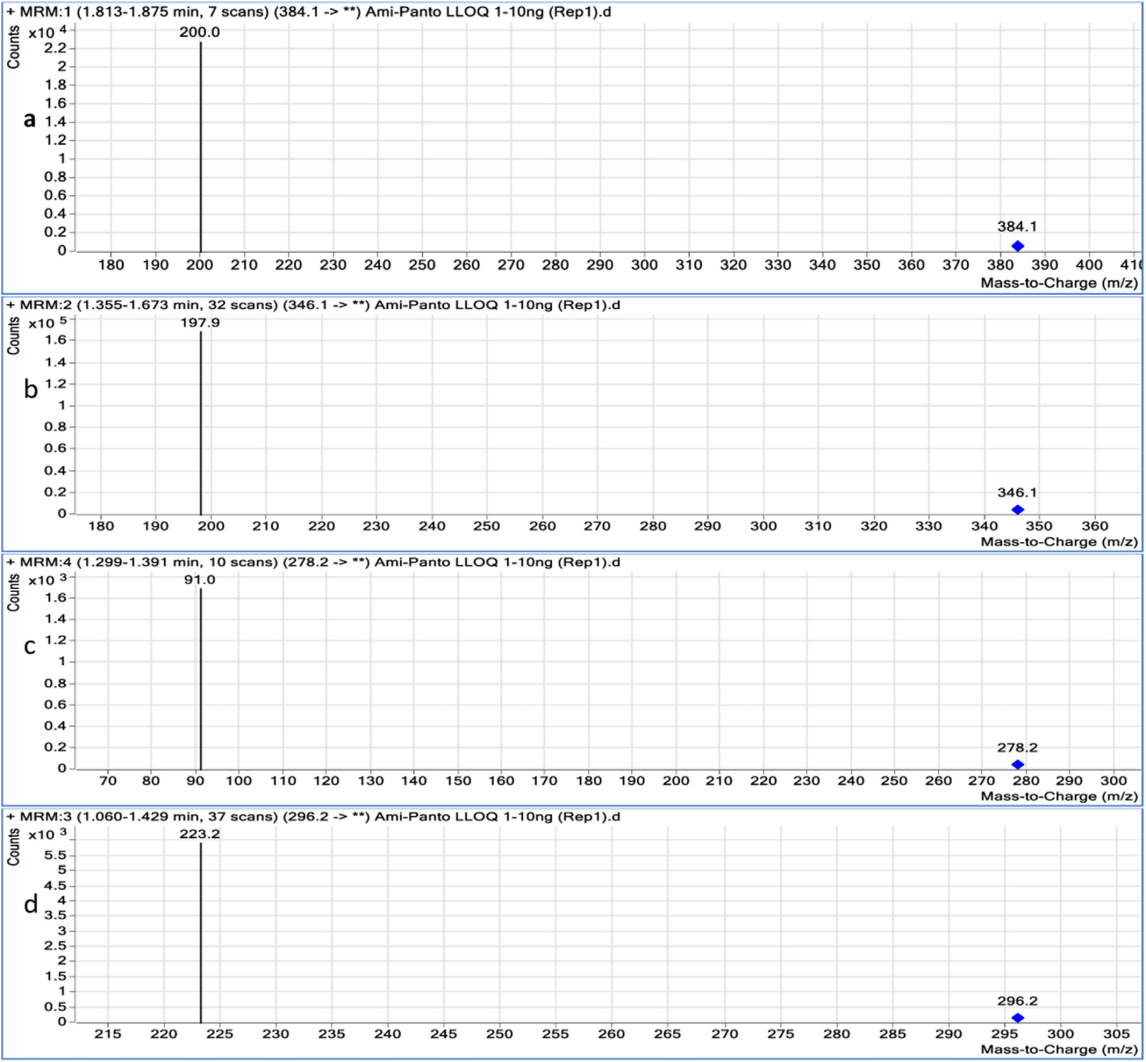
Representative ESI mass spectra scan for the product ion of (a) Pan, (b) Omp, (c) Ami and (d) Dot.

MS/MS parameters were optimized for the analyzed drugs and IS to develop maximum stable and sensitive response with maximum peak area in positive ion mode. The condition was tuned to discover m/z of Q_1_ ion (precursor ion) and m/z of Q_3_ ion (product ion), by using ESI source, the studied drugs were ionized and then detected by multiple reaction monitoring (MRM) mode. The subsequent transitions were monitored m/z 384.1/200, 278.2/91, 346.1/197, 9 and 296.2/223.2 for Pan, Ami, Omp and Dot (IS), respectively (Figure 3).

### 3.2 Bio-analytical method validation (Food and Drug Administration, 2011; Guideline EMEA 192217, 2015)

#### 3.2.1 Linearity

Six calibration curves were applied to assess linearity: a blank sample, a zero sample and 8 non–zero samples covering the predicated range 0.01–4 μgmL^−1^ for Pan and 0.001–0.1 μgmL^−1^ for Ami in rabbit's plasma taking into consideration the reported C _max_ values. For each drug, calibration curves were constructed by sketching peak area ratios (peak area of each drug/peak area of IS) against concentration (C) applying weighting factor 1/x linear regression (Refer to supplementary material Table 4S). The linearity was assessed by computing the mean regression coefficients (r^2^) and by estimating the back calculated concentrations of the calibration standards. The results obtained were less than 20 % deviation with coefficient of variation 9.17 and 0.38 % for Pan and Ami respectively, from the nominal concentration at the LLOQ (lowest concentration of an analyte that can be detected and should be equal to or less than 5 % of the expected C _max_) and less than 15 % deviation at other levels from nominal concentrations.

#### 3.2.2 Selectivity

Six drug free rabbit's plasma samples were selected randomly from various sources to check the interferences of endogenous component that could be found in the matrix and endogenous plasma protein with studied drugs and IS. No significant interference was observed for all the plasma blanks at retention times of the drug and IS.

#### 3.2.3 Extraction recovery

The separation efficiency of analytes from the matrix (rabbit's plasma) is defined by extraction recovery. This was achieved by comparison the mean peak responses of six pre extracted quality control (QC) samples of low, medium and high concentrations (LQC, MQC and HQC) to mean peak responses of six post extracted QC samples. The extraction recovery (ER %) for Pan, Ami and the same for Omp and Dot was computed by dividing the peak response of pre extraction sample by peak response of post extraction sample then multiplied the result by 100. The data revealed that the extraction recoveries were reproducible and indicated good extraction efficiency of the developed method (Refer to supplementary material Table 5S).

#### 3.2.4 Matrix effect

The plasma samples were spiked with analytes after extraction procedure to prepare three levels of QC samples. The mean peak areas were recorded and compared to the mean peak area of standard solutions of the same concentrations of the QC samples and the same for the IS (Omp and Dot).The precentage matrix factor (%MF) of each drug and IS was computed in order to estimate the matrix effect of the method then normalized matrix factor (Normalized-IS MF) was calculted by dividing MF of the analyte to the MF of its IS. The CV% of the IS-normalized MF was in acceptable range, indicating no significance with respect to enhancement effect of the matrix or ion suppression (Refer to supplementary material Table 6S).

#### 3.2.5 Precision and accuracy

Four levels of QCs (LLOQ QC, LQC, MQC and HQC) samples were injected in six replicates for intra-run and on three days for inter run precision and accuracy. Precision was estimated by computing CV% from different determinations. The acceptance criteria of accuracy to be within a range of 85–115 % at LQC, MQC and HQC levels and 80–120 % at LLOQ QC level and CV% (precision) to be within 15 % at LQC, MQC and HQC and 20 % at LLOQ QC level (Table 1).

**Table 1:** Accuracy and precision results for Pan and Ami in rabbit plasma.

QC Samples	Intra-run accuracy and precision				between-run accuracy and precision			
	Mean recovered Conc. (μgmL^−1^)	SD	CV%	% Nominal	Mean recovered Conc. (μgmL^−1^)	SD	CV%	% Nominal
**Pan**	**Pan**							
LLOQ (0.01μgmL^−1^)	0.0101	0.02	0.18	101.00	0.0101	0.05	0.51	101.00
LQC (0.03μgmL^−1^)	0.0293	0.38	1.29	97.70	0.0299	0.47	1.56	99.67
MQC (1.6μgmL^−1^)	1.6078	12.33	0.77	100.49	1.5934	8.31	0.52	99.59
HQC(3 μgmL^−1^)	3.0075	14.20	0.47	100.25	2.9808	2.00	0.07	99.36
**Ami**	**Ami**							
LLOQ (0.001μgmL^−1^)	0.0011	0.02	1.94	110.00	0.0011	0.03	2.62	110.00
LQC (0.003μgmL^−1^)	0.0029	0.05	1.57	96.67	0.0030	0.02	0.63	100.00
MQC (0.03μgmL^−1^)	0.0289	1.12	3.85	96.33	0.0295	0.52	1.77	98.33
HQC (0.075 μgmL^−1^)	0.0756	2.24	2.97	100.80	0.0733	2.96	4.03	97.73

#### 3.2.6 Dilution integrity

The dilution integrity of the proposed method was evaluated by utilization two dilution factors: the plasma sample was spiked with a concentration of 5μgmL^−1^ and 0.16μgmL^−1^ for Pan and Ami respectively. Two groups of six samples of dilution integrity samples were prepared by diluting them (2 and 4 fold). The CV % for 2-fold dilution test was 2.49 and 1.69 with accuracy results 99.64 % and 99.21 % for Pan and Ami respectively. The CV % for 4-fold dilution test was 0.64 and 1.81 with accuracy results 99.90 % and 98.68 % for Pan and Ami respectively.

#### 3.2.7 Stability

QC samples at three concentration levels (LQC, MQC and HQC) were injected in triplicates to evaluate the drugs stability in the plasma samples. The different sets of QC samples prepared using the formly described procedure in “sample prepration”, were exposed to diverse storge conditions and then examined with fresh samples of the same concentration. for short term stability, the QC samples (spiked plasma) were stored at 25 ± 2 °C for 4 h before analysis followed by sample preperation and analysis. For long term stability, the QC samples were stored at -70 ± 10 °C for 20 days followed by sample preperation and analysis. For post-preperative stability study, the processed QC samples were stored in auto sampler at 10 °C for 4 h, then were analyzed. For dry extract stability, the processed QC samples were stored at -70 °C±10 °C for 12 h without reconstitution. For freeze and thaw stability, QC samples were stored at (-70 ± 5 °C) and subjected to three freeze and thaw cycles with minimum freezing time of 12 h and thawed for 2 h at room temperature. The stock sloution stability for each drug concentrations were kept at (−20 ± 5) for 7days. The QC samples were found to be ±15 % within the nominal concentrations through the whole assay showing good stability results as shown in Table 2.

**Table 2:** Summary of stability results of Pan and Ami in human plasma using the proposed LC- MS/MS method.

Stability Term	QC sample	Pan				Ami			
		QC Conc (μgmL^−1^)	Mean recovered Conc (μgmL^−1^)	% Nominal ±SD	CV%	QC Conc (μgmL^−1^)	Mean recoverd Conc (μgmL^−1^)	% Nominal ±SD	CV%
Short term (after 4hr) Bench top stability or	LQCL	0.030	0.0296	98.67 ± 0.32	1.08	0.003	0.0029	96.67 ± 0.03	0.88
	MQC	1.600	1.5978	99.86 ± 0.43	0.21	0.030	0.0293	97.67 ± 0.62	2.13
	HQC	3.000	2.9847	99.49 ± 7.79	0.26	0.075	0.0746	99.46 ± 0.30	0.41
Long term (after 20days)	LQC	0.030	0.0289	96.33 ± 0.16	0.55	0.003	0.0030	100.00 ± 0.02	0.59
	MQC	1.600	1.5837	98.98 ± 9.38	0.59	0.030	0.0290	96.67 ± 0.42	1.44
	HQC	3.000	2.9466	98.22 ± 50.71	1.72	0.075	0.0741	98.80 ± 0.56	0.76
Auto-sampler (post-preperative stability)	LQC	0.030	0.0298	99.38 ± 0.20	0.67	0.003	0.0029	97.29 ± 0.01	0.36
	MQC	1.600	1.5933	99.58 ± 7.90	0.50	0.030	0.0292	97.33 ± 0.20	0.69
	HQC	3.000	2.9901	99.67 ± 3.22	0.11	0.075	0.0738	98.40 ± 0.42	0.56
Freeze- thaw (after three cycles)	LQC	0.030	0.0297	99.11 ± 0.36	1.23	0.003	0.0029	99.33 ± 0.05	1.80
	MQC	1.600	1.5961	99.75 ± 4.81	0.3	0.030	0.0293	97.66 ± 0.18	0.63
	HQC	3.000	2.9932	99.77 ± 5.8	0.19	0.075	0.0741	98.80 ± 0.48	0.65
Dry extract stability	LQC	0.030	0.0287	95.66 ± 1.49	1.55	0.003	0.0028	91.74 ± 1.32	1.44
	MQC	1.600	1.5781	98.63 ± 0.18	0.19	0.030	0.0280	91.74 ± 2.62	2.68
	HQC	3.000	2.9381	97.93 ± 1.05	1.07	0.075	0.0743	96.79 ± 0.22	0.23
Stock stability solution	Pan			97.26	0.51				
	Ami			96.90	0.63				
	Omp (IS)			97.17	0.52				
	Dot (IS)			97.22	0.31				

### 3.3 Micrometric properties

The results of micrometric properties of the prepared formulae, Hausner's ratio values ranged from 1.15 ± 0.06 to 1.36 ± 0.04 indicating low to moderate antiparticle friction (Badawy et al., 2011). Carr's index (%) compressibility ranged from 13.10 ± 4.44 to 26.42 ± 2.13 % and the angle of repose ranged from 28.86^°^ ± 4.67–35.80^°^ ± 1.14 indicating fair flow properties (Shahi et al., 2008) (Refer to supplementary material Table 7S and Figure 1S).

### 3.4 Effervescence cessation time and pH determination

The prepared formulae show low effervescence cessation time (44.00 ± 1.00 to 45.00 ± 1.00 s), due to the low amount of citric acid added to limit the decrease in pH and enhance the stability of Pan. The pH in water was basic for all the prepared formulae while the pH in 0.1N HCl was acidic for F1, F2 and F3 formulae and increased in formulae F4, F5, F6, F7, F8 and F9 (Refer to supplementary material Table 7S and Figure 1S).

### 3.5 Dissolution method validation

A linear relationship between the peak areas and corresponding concentration over the concentration range of 10–50 and 2–12 μgmL^−1^ for Pan and Ami was obtained with good regression coefficients. LOD and LOQ values were found to be 1.095 and 3.318 μgmL^−1^/0.614 and 1.860 μgmL^−1^ for Pan and Ami respectively. Accuracy was expressed in percentage recovery for three different concentrations of Pan and Ami respectively representing 80 %, 100 % and 120 %. Also precision was expressed in 6 times repeatability and robustness by changing the analyst and temperature showed RSD within specified limit (5 %), Table 8S. The placebo of formula F5 was subjected to dissolution test and was analyzed. The specificity of the dissolution test demonstrated no interferences, where no other additional peaks observed (Refer to supplementary material Figure 2S).

### 3.6 In vitro dissolution media

The dissolution profiles of Pan and Ami effervescent granules showed that the release of both was pH dependent (Refer to supplementary material Figure 3S; Table 9S). The increase in pan release and the decrease in the rate of its degradation were attained by increasing the amounts of NaHCO_3_ and Na_2_CO_3_ in the formulae that increased the pH of the dissolution medium, resulted in more stability of Pan. This was achieved in formulae F5, F6, F7, F8 and F9, while the amount of Ami decreased, because it might be absorbed at low pH. So, formula F5 was chosen as a compromise solution, where high stability of Pan in stomach was attained and accordingly, high pharmacological action and the release of Ami was satisfactory. The release achieved by formula F5 was 80.77 ± 0.64 and 90.93 ± 1.36 % for Pan and Ami, respectively. According to Design–Expert® software,statistical analysis of full factorial design (3^2^) for optimization of Pan and Ami effervescent granules showed that the formula F5 provided best dissolution results with 80.77 ± 0.64 and 90.93 ± 1.36 % for Pan and Ami released respectively, and for that, it was chosen for stability and bioavailability study. For additional data for the dissolution of the prepared formula, output data for full factorial design and the summary of ANOVA (Refer to supplementary material Table 10S-11S).

### 3.7 Stability study

**Figure 4:**
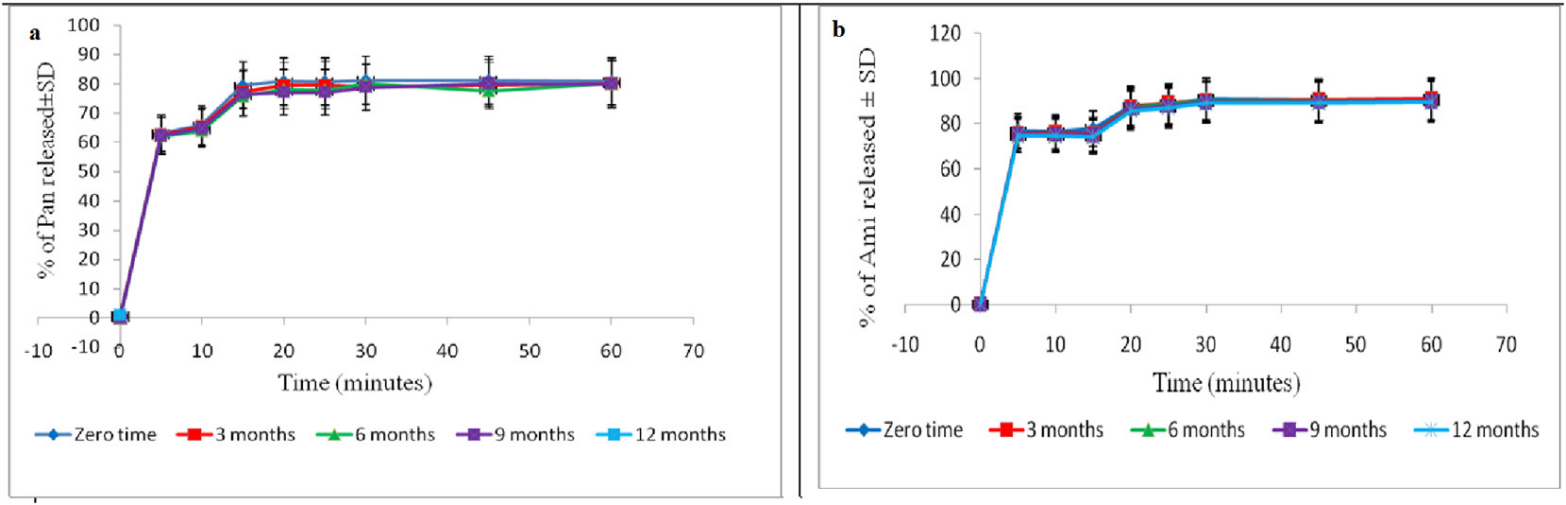
Dissolution profile for (a) Pan and (b) Ami from the selected formula F5 during stability study.

The selected formula F5 showed no noticeable change during stability study in dissolution profile as shown in Figure 4, micrometrics properties, effervescence cessation time and pH in water/0.1N HCl (Refer to supplementary material Figure 4S).

### 3.8 Pharmacokinetic and statistical analysis of data

**Figure 5:**
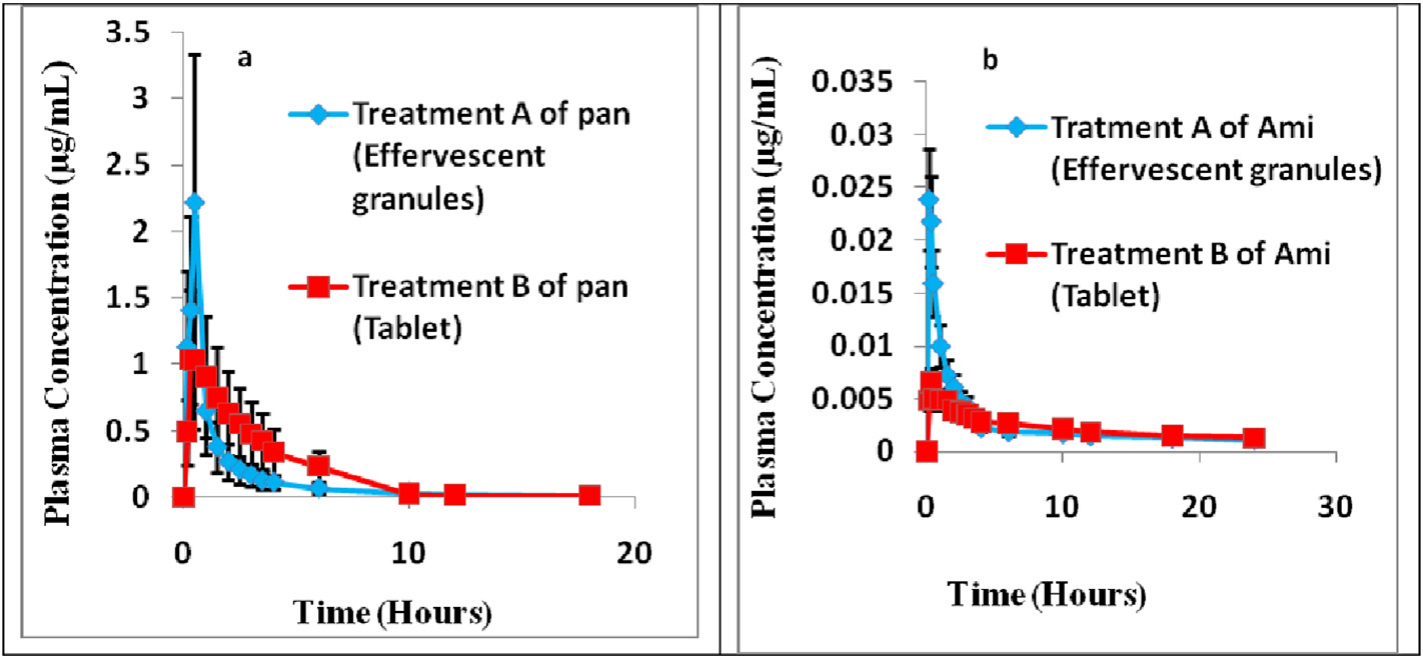
Mean plasma concentration–time profile for (a) Pan and (b) Ami after the oral administration of (Treatment A) and (Treatment B) to 6 healthy rabbits.

Pharmacokinetic parameters of Pan and Ami in Treatment A (effervescent granules) and Treatment B (tablet) were shown in Table 3, then Statistical evaluation of pharmacokinetic parameters was performed applying one way ANOVA, Table 4, it was found that the mean value of maximum plasma concentration for Pan (C_max_) was 2.22 ± 1.57 and 1.30 ± 0.23 μgmL^−1^ for Treatment A and B, respectively Figure 5a. One way ANOVA analysis revealed a significant difference between C_max_ of Treatment A and B as shown in Table 4.This higher C_max_ of Treatment A may be attributed to the decrease in acid degradation of Pan due to the presence of the basic excipients (NaHCO_3_ and Na_2_CO_3_). The half life absorption time t_1/2 abs_ of Treatment A with mean value 0.09 ± 0.01hr was higher than the t_1/2 abs_ of Treatment B with mean value 0.06 ± 0.31 h with significant ANOVA analysis difference (*p*-value = 0.002) meaning that the drug released in plasma was faster in Treatment B than A. Also the elimination half-life time t_1/2 el_ of Treatment A with mean value 4.12 ± 1.00 h was higher than that of Treatment B with mean value 2.43 ± 0.08 h showing significant difference (*p*-value = 0.035). Besides, the apparent volume of distribution Vd/F of treatment A with mean value 5.02 ± 1.81L/Kg was higher than B with mean value 1.92 ± 0.30L/Kg, as shown in Table 3. This could be the result of difference in bioavailability between Treatment A and B formulations.

**Table 3:** Pharmacokinetics parameters results of Pan and Ami in Treatment A and Treatment B.

Pharmacokinetic parameters∗	n = 6					
	Treatment A			Treatment B		
	Mean	SD	CV%	Mean	SD	CV%
	**Formulation (Pan)**					

**Pan = 2.05 mg/kg**					

k _el_ (hr^−1^)	0.17	0.05	26.69	0.29	0.01	3.49
t_1/2 el_ (hr)	4.12	1.00	24.32	2.43	0.08	3.41
AUC _0-24_ (μg.hr/mL)	2.60	0.86	33.10	3.74	0.46	12.31
AUC _0–∞_ (μg.hr/mL)	2.66	0.86	32.32	3.77	0.46	12.19
AUMC_0–∞_(μg.hr^2^/mL)	7.78	0.92	11.83	12.62	1.25	9.90
T _max_ (hr)	0.50	0	0	0.39	0.10	25.71
C _max (_μg/mL)	2.22	1.57	70.65	1.30	0.23	17.71
V_d_/F (L/kg)	5.02	1.81	36.08	1.92	0.30	15.65
CL/F (ml/min/kg)	13.67	3.72	27.22	9.17	1.14	12.43
K _abs_ (hr^−1^)	7.90	0.56	7.15	11.20	0.00	0.00
t _1/2 abs_ (hr)	0.09	0.01	7.42	0.06	0.31	14.63
MRT _0-∞_ (hr)	3.15	1.09	34.65	3.35	0.13	3.68
	**Formulation (Ami)**					

**Ami = 0.51 mg/kg**					

k _el_ (hr^−1^)	0.03	0.01	16.45	0.04	0.01	20.97
t_1/2 el_ (hr)	21.59	3.34	15.47	17.50	3.44	19.65
AUC _0-24_ (μg.hr/mL)	0.06	0.02	36.36	0.05	0.01	16.98
AUC _0–∞_ (μg.hr/mL)	0.10	0.02	21.42	0.09	0.02	25.84
AUMC_0–∞_ (μg.hr^2^/mL)	2.38	0.45	18.75	2.22	1.00	45.01
T _max_ (hr)	0.22	0.10	43.11	0.72	0.67	93.32
C _max (_μg/mL)	0.03	0.01	40.41	0.01	0.002	23.81
V_d_/F (L/kg)	166.37	52.45	31.53	145.87	31.22	21.40
CL/F (mL/min/kg)	87.95	19.74	22.45	97.11	21.85	22.50
K _abs_ (hr^−1^)	12.86	7.17	55.79	3.41	2.02	59.08
t _1/2 abs_ (hr)	0.08	0.06	82.30	0.31	0.28	89.67
MRT _0-∞_ (hr)	25.49	6.92	27.16	24.46	4.83	24.47

∗ Parameters are K _el_: Elimination rate constant, t_1/2, el_:Elimination half-life of drug, AUC_0–24_: Area under the curve up to 24 h, AUC_**0-∞**_: Area under the curve up to infinity, AUMC_**0-∞:**_Area under the first moment curve up to infinity, T _max_: Maximum plasma concentration, C _max_: maximum plasma concentration, V_d_: Apparent volume distribution of drug, F: bioavailability of drug, CL: Clearance,. K _abs_: Absorption rate constant, t _½ abs_: Absorption half-life, MRT_0–**∞**_: Mean residual time up to infinity, (n = 6, no of rabbit in each group).

**Table 4:** Summary of ANOVA test for pharmacokinetic parameters following the administration of Treatment A and Treatment B.

Variables	DF	SS	MS	F -calculated	P-value	F-Critical	
	**Formulation (Pan)**						
k_el_	1	0.040659	0.040659452	47.48512287	0.0000424093	4.964602701	S
t_1/2 el_	1	9.990031718	9.990032	24.57396416	0.000572387	4.964602701	S
AUC _0-24_	1	1.942226	1.942225753	4.100431315	0.00785936	4.964602701	NS
AUC _0–∞_	1	1.8542499	1.8542499	3.898	0.120	4.964602701	NS
AUMC_0–∞_	1	4433.437	4433.437033	3.414577326	0.094375974	4.964602701	NS
T _max_	1	0.009263	0.009262963	2.5	0.1449276	4.964602701	NS
C _max_	1	4.014852279	4.014852279	5.215636	0.048	4.964602701	S
V_d_/F	1	28.80014	28.80014377	21.39541761	0.000942605	4.964602701	S
CL/F	1	60.76912	60.769122	10.03318166	0.010029824	4.964602701	S
K _abs_	1	32.66357	32.66356532	87.93778631	2.85561E-06	4.964602701	S
t _1/2 abs_	1	0.0010279	0.0010279	48.039	0.002	4.964602701	S
MRT	1	0.128107	0.128106881	0.265808202	0.617362756	4.964602701	NS
	**Formulation (Ami)**						

k _el_	1	0.000195	0.0001952	4.792314491	0.053413976	4.964602701	NS
t_1/2 el_	1	25.09465	25.094651	2.184361437	0.213489081	4.964602701	NS
AUC _0-24_	1	0.00016	0.0001598	0.68422833	0.42743197	4.964602701	NS
AUC _0–∞_	1	0.000214	0.0002139	0.565413106	0.469415754	4.964602701	NS
AUMC_0–∞_	1	0.082142	0.0821424	0.171936615	0.687151419	4.964602701	NS
T _max_	1	0.705966	0.705966	4.106494527	0.070219993	4.964602701	NS
C _max_	1	0.001051	0.001051	23.53372713	0.000670214	4.964602701	S
V_d_/F	1	1259.87	1259.8769	0.845427289	0.379494236	4.964602701	NS
CL/F	1	251.8523	251.85225	0.726044291	0.414109041	4.964602701	NS
K _abs_	1	267.7367	267.73671	12.05532912	0.006000196	4.964602701	S
t _1/2 abs_	1	0.168785	0.1687854	9.659051581	0.011100273	4.964602701	S
MRT	1	3.223482	3.2234821	0.113052778	0.743643785	4.964602701	NS

DF: Degree of freedom, SS: Sum of Squares, MS: Mean of Squares, NS: Non significant difference, S: Significant difference.

Although the release of Ami acquired marked decreased at high pH values but it was not affected by administering the chosen formula. The release of Ami was higher in the Treatment A than B, whereas the mean value of C_max_ for Ami was 0.03 ± 0.01 and 0.01 ± 0.002 μgmL^−1^ for Treatment A and B, respectively, Figure 5b. This most likely due to the way of dispensing, where Treatment A was given to rabbits in solution while B as suspended particles, that would affect the release of Ami. Also the mean value of t_1/2abs_ for Ami was 0.08 ± 0.06 and 0.31 ± 0.28 h for Treatment A and B, respectively as shown in Table 3. While t_1/2el_ and V_d_/F of Treatment A with mean value 21.59 ± 3.34 h and 166.37 ± 52.45 L/kg, respectively showed no significance when compared with those of Treatment B with mean values 17.50 ± 3.44 h and 145.87 ± 31.22 L/kg, respectively, Table 4.

## 4 Conclusion

The developed LC-MS/MS was applied for sensitive synchronous determination of combination of PPI drug (Pan) with tri-cyclic antidepressant (Ami) in rabbit's plasma with acceptable results according to EMA bioanalytical validation guidelines. Moreover, liquid-liquid extraction procedure was simple, fast and easy to be applied in all laboratories rather than the solid phase extraction procedure. In addition short run time was achieved (2.2 min) and the low value of LLOQ making it successful for application in pharmacokinetic study. For previous reasons the proposed method was satisfactory applied in bioequivalence and for pharmacokinetic parameters comparison between novel effervescent granules formulation contained Pan and Ami with their marketing tablet form, Zurcal® and Tryptizol® in rabbit plasma following their oral administration.

## Declarations

### Author contribution statement

Asmaa A. El Zaher; Ehab F. El Kady: Conceived and designed the experiments; Analyzed and interpreted the data; Wrote the paper.

Hussein M. EL Messiry: Conceived and designed the experiments; Performed the experiments; Wrote the paper.

Hind E. El Ghwas: Conceived and designed the experiments; Performed the experiments; Analyzed and interpreted the data; Contributed reagents, materials, analysis tools or data; Wrote the paper.

Ola M. El Houssini: Conceived and designed the experiments; Analyzed and interpreted the data; Contributed reagents, materials, analysis tools or data; Wrote the paper.

### Funding statement

This research did not receive any specific grant from funding agencies in the public, commercial, or not-for-profit sectors.

### Data availability statement

Data included in article/supplementary material/referenced in article.

### Declaration of interests statement

The authors declare no conflict of interest.

### Additional information

No additional information is available for this paper.

## Appendix A Supplementary data

2-8-HY-SM2_(2) 2-8-HY-SM2_(2)
