# Supplementary material for: Synchronous LC-MS/MS determination of pantoprazole and amitriptyline in rabbit plasma: application to comparative in vivo pharmacokinetic study of novel formulated effervescent granules with its marketed tablet dosage form
Source: Heliyon. 2021 Aug 10;7(8):e07752. doi: 10.1016/j.heliyon.2021.e07752 (PMC8375633; doi:10.1016/j.heliyon.2021.e07752)
Supplement: 2-8-HY-SM2_(2) [file mmc1.doc]

**Table 1S: The performance characteristics of the proposed LC-MS/MS chromatographic method and the published methods**

|  | **Mobile Phase** | **Stationary phase** | **Sample preparation** | **Application/Remarks** | **Ref .No** |
| --- | --- | --- | --- | --- | --- |
| 1 | Acetonitrile: water: methanol (57:25:18, v/v/v) containing 10 mmol/l acetic acid + 20 mmol/l ammonium acetate) at 0.7 mL min^-1^ in isocratic elution mode | Genesis C_8_ 4 µm at 40°C | liquid/liquid extraction using diethyl-ether/dichloromethane | Determination of Pan using lansoprazole as internal standard (IS) in human plasma (run time 4.5 min) | Peres et al., 2004 |
| 2 | Methanol: water (60:40, v/v), each containing 1% of 5 mol L^-1^ ammonium acetate) at a flow rate of 0.3 mLmin^-1^ in isocratic elution mode | Rp column at 40°C | liquid–liquid extraction with methylene chloride and tert-butyl ether, protein precipitation with methanol) | Determination of Pan using omeprazole as IS in human plasma (run time 2.2 min) | Li et al., 2011 |
| 3 | 10 mM ammonium acetate (pH 7.10): acetonitrile (30:70, v/v) with flow rate at 0.6 mLmin^-1^ in isocratic elution mode | Zorbax SB-C_18_ at 30°C | Protein precipitation using acetonitrile | Determination of Pan using Pan D3(PSD3) as IS in human plasma (run time 1.34 min) | Challa et al., 2010 |
| 4 | 100 mM ammonium acetate: acetonitrile (6:94, v/v) with flow rate at 0.4 mLmin^-1^ in isocratic elution mode | Hypersil silica at 40°C | liquid/liquid extraction using tert-butyl methyl and 0.5M sodium hydroxide as alkali solution | Determination of Ami and nortriptyline using imipramine as IS in human plasma (run time 8.5 min) | Kudo et al., 1997 |
| 5 | 10 mM ammonium formate: acetonitrile: methanol (20:40:40, v v) at a flow rate 0.8 mLmin^-1^ in isocratic elution mode | C_18_ INERTSIL at 40°C | Protein precipitation using acetonitrile | Determination of Pan, lansoprazole esomeprazole, and rabeprazole in human plasma using esitalopram as IS (run time 3.5 min) | Elkady et al., 2018 |
| 6 | Water and acetonitrile with 0.1% formic acid in gradient elution mode | Hypersil Gold C_18_ at 25°C. | solid-phase extraction (SPE) column | Determination of Ami, nortriptyline, desipramine, and imipramine in human plasma and Ami-D3, nortriptyline-D3, desipramine-D3 and imipramine-D3 as IS (run time 3.5 min) | Breaud et al., 2010 |
| 7 | (0.1% formic acid in water) and phase B2 (0.1% formic acid in acetonitrile) with flow rate 0.2 mLmin^-1^ in gradient elution mode | X Terra MS C_18_ 25 | Protein precipitation using acetonitrile | Determination of a Ami, nortriptyline, desipramine, imipramine and selective serotonin reuptake inhibitors and Mianserin-D3, amitriptyline-D6, clomipramine-D3, and imipramine-D3 in human serum ( run time 6 min) | Sauvage et al., 2006 |
| 8 | Acetonitrile: water (90:10, v/v) with flow rate 0.6 mLmin^-1^ in isocratic elution mode | Symmetry C_18_ at 25°C | Solid phase extraction with SPE cartridge | Determination of Pan and IS in human urine (run time 4 min) | Bhaskara et al., 2015 |
|  | Acetonitrile: 4mM ammonium acetate solution (comprising 0.05% formic acid) (40:60, v/v) with flow rate of 0.6 mLmin^-1^ in isocratic elution mode | Gemini Phenomenex column C_18_ at 25°C | Liquid-Liquid extraction method with diethyl ether: dichloromethane (70:30, v/v) | Determination of Pan and Ami using Dot and Omp as in rabbit plasma (run time 2.1 min) | Present work |

**Table 2S: The detailed compositions of each effervescent granule formulation encountered in study design and Output data of general factorial design (3^2^) for optimization the new combination of each formula:**

| **Exp** | **Pan**  **(mg)** | **Ami**  **(mg)** | **Citric acid**  **(mg)** | **PEG**  **4000** | **Vanillin** | **Na_2_CO_3_**  **(mg)** | **NaHCO_3_**  **(mg)** | **% Pan released**  **after 60 min** | **% Ami released after 60 min** | **Desirability** |
| --- | --- | --- | --- | --- | --- | --- | --- | --- | --- | --- |
| **F1** | 40 | 10 | 100 | 5% | 0.3% | 200 | 2000 | 16.93±0.64 | 97.20±1.65 | 0.081 |
| **F2** | 40 | 10 | 100 | 5% | 0.3% | 600 | 2000 | 21.62±1.80 | 97.89±0.69 | 0.273 |
| **F3** | 40 | 10 | 100 | 5% | 0.3% | 800 | 2000 | 23.97±0.72 | 97.30±0.75 | 0.326 |
| **F4** | 40 | 10 | 100 | 5% | 0.3% | 200 | 5800 | 40.08±2.00 | 97.65±0.49 | 0.581 |
| ***F5 (selected)** | 40 | 10 | 100 | 5% | 0.3% | 600 | 5800 | 80.77±0.64 | 90.93±1.36 | **0.841** |
| **F6** | 40 | 10 | 100 | 5% | 0.3% | 800 | 5800 | 81.07±0.70 | 87.33±0.85 | 0.772 |
| **F7** | 40 | 10 | 100 | 5% | 0.3% | 200 | 8500 | 80.37±2.61 | 80.04±0.51 | 0.568 |
| **F8** | 40 | 10 | 100 | 5% | 0.3% | 600 | 8500 | 81.84±2.11 | 73.33±3.46 | 0.453 |
| **F9** | 40 | 10 | 100 | 5% | 0.3% | 800 | 8500 | 83.13±1.00 | 69.02±0.44 | 0.129 |

**Table 3S: General factorial experimental design (3^2^) for optimization of the prepared formulae:**

| **Factors** | **Levels** | | |
| --- | --- | --- | --- |
| X_1_: Na_2_CO_3_ | 200 | 600 | 800 |
| X_2_: NaHCO_3_ | 2000 | 5800 | 8500 |
| **Responses** | **Constraints** | | |
| Y_1_: percentage of **Pan** release after 60min | Maximize | | |
| Y_2_: percentage of **Ami** release after 60min | Maximize | | |

**Table 4S: Linearity results of Pan and Ami in rabbit plasma:**

| **Calibration parameters** | **Pan** | **Ami** |
| --- | --- | --- |
| **Calibration curve range (µgmL^-1^)** | 0.01-4 | 0.001-0.1 |
| **Lower limit of quantification (LLOQ) (µgmL^-1^)** | 0.01 | 0.001 |
| **Retention time (t_R_) (min)** | 1.85 | 1.41 |
| **Mean regression**  **coefficient** | 0.9961 | 0.9990 |
| **Mean intercept** | 8.2931x10^-4^ | 0.0772 |
| **Mean slope** | 3.7589x10^-4^ | 0.0201 |

**Table 5S: Recovery extraction results of Pan and Ami from rabbit plasma using the proposed LC-MS/MS method:**

| **QC sample** | **Pan** | | **Omp (IS)** | | **QC sample** | **Ami** | | **Dot (IS)** | | **n** |
| --- | --- | --- | --- | --- | --- | --- | --- | --- | --- | --- |
|  | **Mean recovery (%)** | **CV%** | **Mean recovery (%)** | **CV%** |  | **Mean recovery**  **(%)** | **CV**  **%** | **Mean recovery (%)** | **CV**  **%** |  |
| **LQC**  **0.03**  **µgmL^-1^** | 100.24 | 0.55 | 99.46 | 1.53 | **LQC**  **0.003**  **µgmL^-1^** | 97.82 | 5.31 | 99.65 | 2.37 | **6** |
| **MQC 1.6**  **µgm L^-1^** | 98.38 | 0.97 | 96.33 | 1.08 | **MQC**  **0.03**  **µgmL^-1^** | 97.13 | 4.57 | 96.19 | 4.29 | **6** |
| **HQC**  **3**  **µgm L^-1^** | 99.36 | 2.02 | 96.45 | 3.10 | **HQC**  **0.075**  **µgmL^-1^** | 95.92 | 1.80 | 97.24 | 1.32 | **6** |

C.V.: Coeﬃcient of variation; n: number of determination.

**Table 6S: Matrix effect results of Pan and Ami in rabbit plasma using the propsed LC-MS/MS method:**

| **Analyte** |  | | | | | | | | | | | | | | | |  | | |
| --- | --- | --- | --- | --- | --- | --- | --- | --- | --- | --- | --- | --- | --- | --- | --- | --- | --- | --- | --- |
|  | **Mean analyte matrix facto**r | | | **CV% of analyte matrix factor** | | | | **Mean IS matrix facto**r | | | **CV% of analyte matrix factor** | | | **Mean normalized matrix factor** | | | **CV% of normalized matrix factor** | | |
|  | **LQC** | **MQC** | **HQC** | **LQC** | **MQC** | **HQC** | **LQC** | | **MQC** | **HQC** | **LQC** | **MQC** | **HQC** | **LQC** | **MQC** | **HQC** | **LQC** | **MQC** | **HQC** |
| **Pan** | 98.86 | 99.81 | 95.70 | 0.91 | 0.75 | 2.80 | 98.06 | | 95.33 | 97.19 | 0.96 | 3.07 | 2.77 | 100.83 | 104.77 | 98.47 | 1.54 | 2.92 | 0.38 |
| **Ami** | 99.63 | 98.46 | 96.54 | 0.83 | 4.63 | 5.34 | 99.35 | | 100.46 | 97.27 | 1.27 | 5.21 | 4.89 | 100.29 | 98.04 | 99.23 | 1.06 | 0.62 | 0.97 |

**Table 7S: Micrometric properties, effervescence cessation time, pH in water and in 0.1N HCl:**

| **Formula** | **Bulk density**  **(P_bulk_) gml^-1^** | **Tapped density (P_tab_) gml^-1^** | **Carr's index (% compressibility)** | **Hausner ̓s Ratio** | **Angle of repose (degrees)** | **Effervescence cessation time (sec)** | **pH in 0.1N HCL** | **pH in water** |
| --- | --- | --- | --- | --- | --- | --- | --- | --- |
| **F1** | 0.36±  0.03 | 0.46±  0.03 | 22.52±  0.70 | 1.29±  0.01 | 28.86 ±  4.67 | 44.00±  1.00 | 3.5± 0.30 | 8.70 ±0.13 |
| **F2** | 0.34±  0.02 | 0.47±  0.03 | 26.42±  2.13 | 1.36±  0.04 | 32.78 ±  3.56 | 45.00±  1.00 | 3.8± 0.10 | 8.85 ±0.07 |
| **F3** | 0.38±  0.02 | 0.48±  0.02 | 21.06±  3.53 | 1.27±  0.06 | 35.49 ±  3.99 | 44.00±  2.00 | 4.0± 0.20 | 8.93 ±0.12 |
| **F4** | 0.34±  0.05 | 0.41±  0.05 | 18.76±  2.43 | 1.23±  0.04 | 33.65 ±  1.42 | 44.00±  1.00 | 6.8 ±0.30 | 9.11 ±0.27 |
| **F5** | 0.33±  0.04 | 0.43±  0.06 | 22.81±  2.16 | 1.29±  0.04 | 34.77 ±  1.70 | 45.00±  0.17 | 7.3 ±0.26 | 9.29 ±0.13 |
| **F6** | 0.37±  0.00 | 0.46±  0.01 | 17.68±  0.87 | 1.21±  0.01 | 35.66 ±  3.91 | 44.00±  1.10 | 7.5 ±0.20 | 9.51 ±0.15 |
| **F7** | 0.35±  0.05 | 0.40±  0.05 | 13.59±  4.19 | 1.16±  0.06 | 34.47 ±  2.33 | 44.00±  1.73 | 8.0 ±0.10 | 9.41 ±0.16 |
| **F8** | 0.35±  0.02 | 0.45±  0.06 | 20.27±  7.36 | 1.26±  0.11 | 31.10±  4.08 | 45.00±  1.00 | 8.5 ±0.40 | 9.31± 0.16 |
| **F9** | 0.36± 0.04 | 0.41± 0.05 | 13.10±  4.44 | 1.15±  0.06 | 35.80 ±  1.14 | 45.00±  0.72 | 8.8 ±0.20 | 9.21± 0.16 |

**Table 8S: Validation parameters and results obtained by dissolution validation proposed HPLC methods for the simultaneous determination of Pan and Ami:**

| **Parameter** | **Pan** | **Ami** |
| --- | --- | --- |
| **Wavelength of detection (nm)** | 230 | 230 |
| **Retention time (t_r_) (min)** | 4.814±0.2 | 11.451±0.2 |
| **Linearity range (µgmL^-1^)** | 10-50 | 2-12 |
| **LOD(µgmL^-1^)** | 1.095 | 0.614 |
| **LOQ(µgmL^-1)^** | 3.318 | 1.860 |
| **Regression coefficient (r^2^)** | 0.9997 | 0.9983 |
| **Slope(b)** | 42.339 | 42.106 |
| **Intercept (a)** | 35.226 | 7.437 |
| **Standard deviation of slope(S_b_)** | 0.424 | 1.01 |
| **Confidence limit of slope** | 42.339±1.00 | 42.106±2.39 |
| **Standard deviation of intercept (Sa)** | 14.050 | 7.83 |
| **Confidence limit of intercept** | 3.2923±39.89 | 7.437±105.28 |
| **Standard error of estimation** | 13.396 | 8.384 |
| ****precision (mean±%RSD)** | 98.93±0.47 | 99.31±0.53 |
| **Drug in bulk (mean±SD)** | 99.81±0.51 | 98.79±0.06 |

** The precision (n=6), average of target concentration of sample (40µgmL^-1^) of Pan and (10 µgmL^-1^) of Ami

**Table 9S: In-vitro release of Pan and Ami from the prepared formulae in dissolution study:**

| **Time (min)** | **Drug** | **F1** | **F2** | **F3** | **F4** | **F5** | **F6** | **F7** | **F8** | **F9** |
| --- | --- | --- | --- | --- | --- | --- | --- | --- | --- | --- |
| **5** | **Pan** | 7.54±  0.79 | 15.32±  2.47 | 18.74±  1.90 | 31.32±  2.19 | 63.11±  3.05 | 62.60±  2.55 | 65.24±3.03 | 67.71±  2.73 | 64.10±  1.37 |
|  | **Ami** | 90.49±  0.82 | 90.48±  2.54 | 86.96±  1.58 | 88.81±  2.01 | 76.83±  1.30 | 65.37±  1.51 | 61.03±  1.43 | 52.77±  1.89 | 52.40±  2.95 |
| **10** | **Pan** | 18.36±  1.47 | 19.49±  1.04 | 20.00±  2.04 | 35.08±  3.21 | 65.77±  0.10 | 65.48±  4.37 | 66.67±  2.02 | 65.24±  1.47 | 64.48±  2.26 |
|  | **Ami** | 93.63±  0.79 | 91.08±  3.04 | 91.36±  2.53 | 93.28±  0.90 | 76.30±  1.40 | 64.58±  1.15 | 62.37±  1.33 | 52.94±  1.78 | 54.10±  3.63 |
| **15** | **Pan** | 16.90±  1.21 | 20.18±  2.28 | 22.75±  1.14 | 39.90±  3.17 | 79.37±  2.48 | 74.96±  0.34 | 74.05±  0.62 | 79.16±  2.21 | 81.24±  1.16 |
|  | **Ami** | 94.92±  0.73 | 93.97±  3.94 | 93.33±  2.35 | 94.15±  1.39 | 77.82±  0.75 | 78.52±  1.95 | 74.81±  3.11 | 86.21±  1.17 | 66.40±  1.45 |
| **20** | **Pan** | 18.09±  1.67 | 20.32±  1.42 | 22.91±  0.59 | 40.23±  2.37 | 80.82±  0.98 | 77.20±  0.46 | 73.08±  1.97 | 79.68±  2.14 | 83.02±  0.51 |
|  | **Ami** | 94.44±  0.22 | 96.35±  0.59 | 95.64±  1.01 | 95.95±  0.35 | 87.76±  0.15 | 85.69±  0.97 | 76.46±  3.23 | 68.07±  0.62 | 66.09±  1.94 |
| **25** | **Pan** | 18.03±  1.49 | 19.56±  1.14 | 23.32±  0.74 | 38.66±  2.13 | 80.72±  0.97 | 77.28±  1.64 | 76.51±  0.72 | 80.21±  1.47 | 82.77±  1.14 |
|  | **Ami** | 95.44±  0.22 | 97.02±  1.52 | 95.86±  1.93 | 97.77±  1.01 | 88.77±  1.58 | 86.36±  1.36 | 77.80±  2.49 | 69.07±  0.38 | 66.59±  1.56 |
| **30** | **Pan** | 19.00±  1.07 | 19.76±  2.05 | 23.30±  1.53 | 40.57±  1.87 | 81.16±  0.62 | 77.27±  1.75 | 79.37±  1.13 | 79.71±  1.02 | 82.65±  1.64 |
|  | **Ami** | 95.48±  1.06 | 96.27±  1.66 | 96.63±  1.05 | 98.32±  0.66 | 90.89±  1.13 | 86.37±  2.16 | 78.97±  1.21 | 70.05±  0.38 | 66.02±  2.40 |
| **45** | **Pan** | 18.03±  0.56 | 20.30±  2.61 | 23.78±  0.99 | 40.68±  1.96 | 81.16±  1.14 | 79.16±  1.85 | 79.36±  1.54 | 80.34±  2.67 | 83.53±  0.84 |
|  | **Ami** | 95.83±  0.57 | 96.50±  1.06 | 96.66±  0.99 | 97.90±  0.58 | 90.59±  1.21 | 87.15±  2.09 | 78.74±  1.84 | 72.13±  2.27 | 69.08±  0.61 |
| **60** | **Pan** | 16.93±  0.64 | 21.62±  1.80 | 23.97±  0.72 | 40.08±  2.00 | 80.77±  0.64 | 81.07±  0.7 | 80.37±  2.61 | 81.84±  2.11 | 83.13±  1.00 |
|  | **Ami** | 97.20±  1.65 | 97.89±  0.69 | 97.30±  0.75 | 97.65±  0.49 | 90.93±  1.36 | 87.33±  0.85 | 80.04±  0.51 | 73.33±  3.46 | 69.02±  0.44 |

**Table 10S: Output data of the full factorial design (3^2^) for optimization of prepared formula:**

| **Responses** | **% Pan released ±SD** | **% Ami released ±SD** |
| --- | --- | --- |
| **Minimum** | 16.93±0.64 | 69.02±0.44 |
| **Maximum** | 83.13±1.00 | 97.89±0.69 |
| **Ratio** | 4.91 | 1.42 |
| **R^2^** | 0.9085 | 0.9848 |
| **Adequate precision** | 22.008 | 46.997 |
| **Adjusted R2** | 0.8965 | 0.9820 |
| **Predicted R2** | 0.8700 | 0.9765 |
| **Significant factors** | A, B and A^2^ | A, B, AB and A^2^ |

**Table 11S: Summary of ANOVA results for the % Pan and% Ami released after 60 min:**

| **Source** | **1Drug** | **SS** | **DF** | **MS** | **F Value** | **P-value** | **Significance** |
| --- | --- | --- | --- | --- | --- | --- | --- |
| **Model** | **Pan** | 19677.21 | 3 | 6559.07 | 76.08 | <0.0001 | **Significant** |
|  | **Ami** | 2870.56 | 5 | 574.11 | 315.93 | < 0.0001 | **Significant** |
| **A(NaHCO_3_)** | **Pan** | 16653.13 | 1 | 16653.13 | 193.17 | 0.0001 |  |
|  | **Ami** | 2173.53 | 1 | 2173.53 | 1196.07 | < 0.0001 |  |
| **B(Na_2_CO_3_)** | **Pan** | 1481.67 | 1 | 1481.67 | 17.19 | 0.0004 |  |
|  | **Ami** | 204.21 | 1 | 204.21 | 112.37 | < 0.0001 |  |
| **A^2^** | **Pan** | 704.29 | 1 | 704.29 | 8.17 | 0.0089 |  |
|  | **Ami** | 364.66 | 1 | 364.66 | 200.67 | < 0.0001 |  |
| **AB** | **Pan** | - | - | - | - | - |  |
|  | **Ami** | 98.55 | 1 | 98.55 | 54.23 | < 0.0001 |  |
| **Residual** | **Pan** | 1982.83 | 23 | 86.21 |  |  |  |
|  | **Ami** | 38.16 | 21 | 1.82 |  |  |  |
| **Lack of fit** | **Pan** | 1937.72 | 5 | 387.54 | 154.63 | <0.0001 | **Significant** |
|  | **Ami** | 23.38 | 3 | 7.79 | 9.49 | <0.0006 | **Significant** |
| **Pure Error** | **Pan** | 45.11 | 18 | 2.51 |  |  |  |
|  | **Ami** | 14.78 | 18 | 0.8213 |  |  |  |
| **Cor Total** | **Pan** | 21660.04 | 26 |  |  |  |  |
|  | **Ami** | 2908.72 | 26 |  |  |  |  |


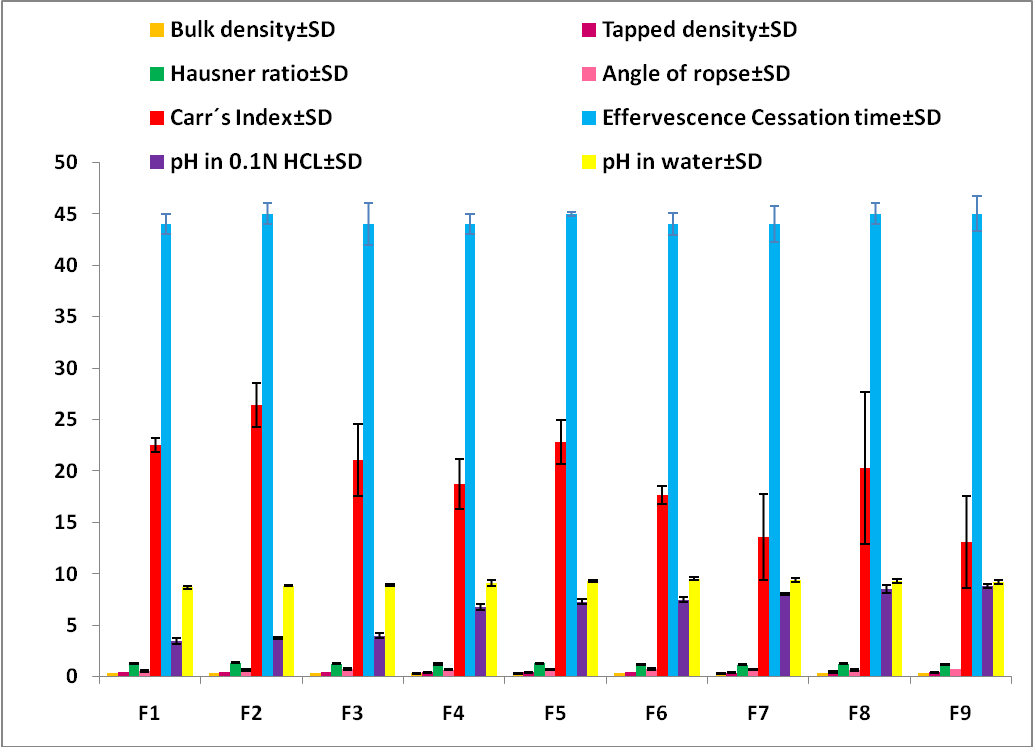


**Fig.1S. Bulk density, Tapped density, Hausner’s ratio and Angle of repose, Carr´s index, Effervescent Cessation time, pH in 0.1N HCL, pH in water for nine effervescent granules:**

| 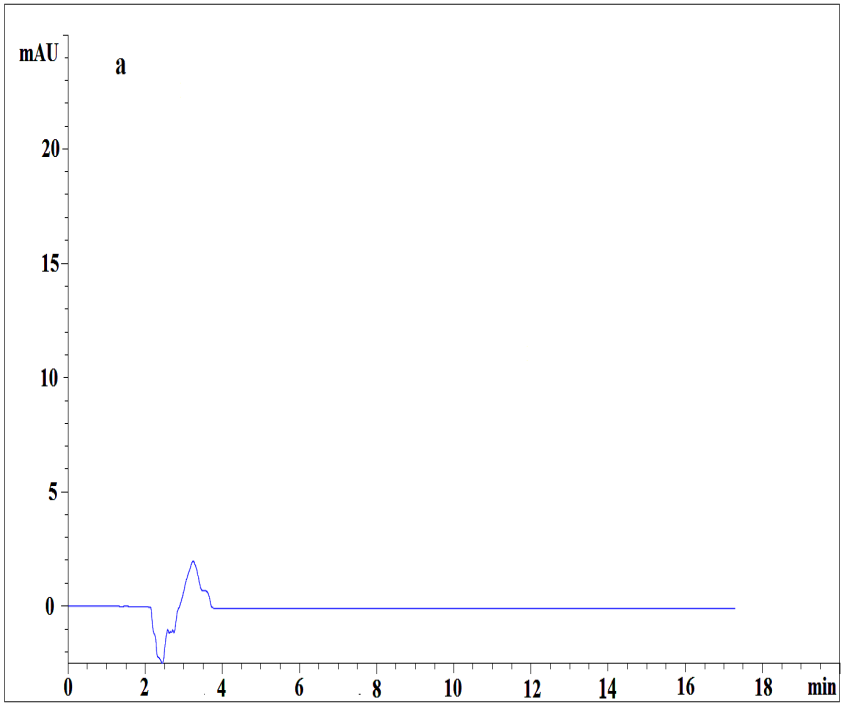 | 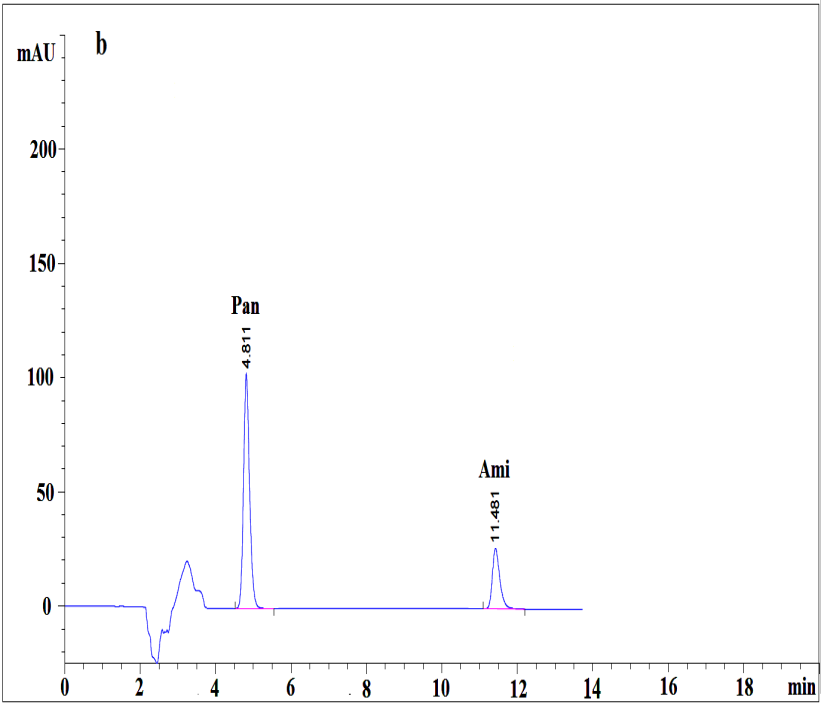 |
| --- | --- |

**Fig.2S.Typical RP-LC chromatogram of (a) placebo solution without active ingredient after dissolution with 0.1N HCl (b) spiked placebo with Pan and Ami (40 and 10 µgmL^-1^ respectively) in 0.1N HCl:**

| ***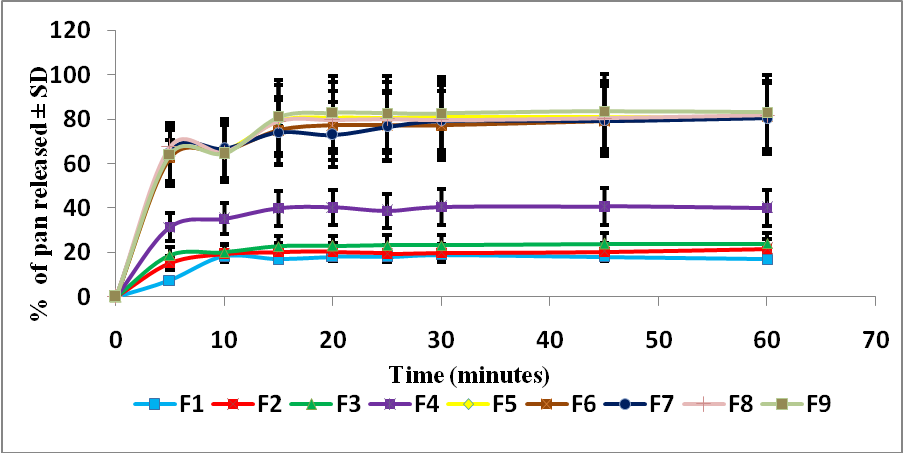***  **a** |
| --- |
| ***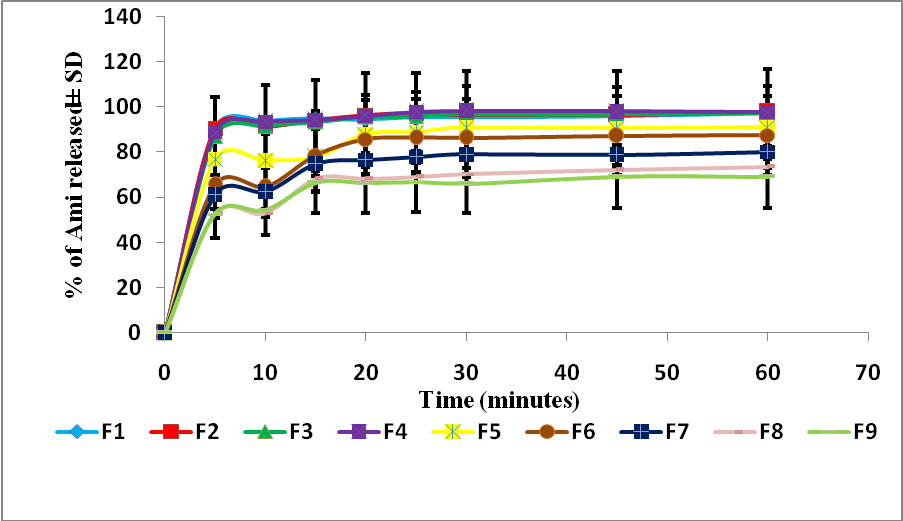***  **b** |

**Fig.3S. In -vitro release profile of (a) Pan and (b) Ami from the prepared formulae:**


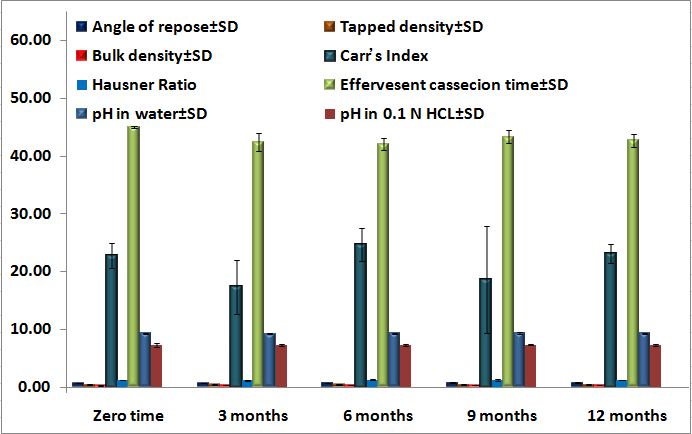


**Fig.4S. Micrometric properties, effervescence cessation time and pH in water/0.1N HCl for formula F5 during stability period:**
